# A Systems Biology Overview on Human Diabetic Nephropathy: From Genetic Susceptibility to Post-Transcriptional and Post-Translational Modifications

**DOI:** 10.1155/2016/7934504

**Published:** 2015-12-20

**Authors:** Francesca Conserva, Loreto Gesualdo, Massimo Papale

**Affiliations:** ^1^Division of Nephrology, Department of Emergency and Organ Transplantation, University of Bari, 70124 Bari, Italy; ^2^Division of Cardiology and Cardiac Rehabilitation, “S. Maugeri” Foundation, IRCCS, Institute of Cassano Murge, 70020 Cassano delle Murge, Italy; ^3^Molecular Medicine Center, Section of Nephrology, Department of Medical and Surgical Sciences, University of Foggia, 71122 Foggia, Italy

## Abstract

Diabetic nephropathy (DN), a microvascular complication occurring in approximately 20–40% of patients with type 2 diabetes mellitus (T2DM), is characterized by the progressive impairment of glomerular filtration and the development of Kimmelstiel-Wilson lesions leading to end-stage renal failure (ESRD). The causes and molecular mechanisms mediating the onset of T2DM chronic complications are yet sketchy and it is not clear why disease progression occurs only in some patients. We performed a systematic analysis of the most relevant studies investigating genetic susceptibility and specific transcriptomic, epigenetic, proteomic, and metabolomic patterns in order to summarize the most significant traits associated with the disease onset and progression. The picture that emerges is complex and fascinating as it includes the regulation/dysregulation of numerous biological processes, converging toward the activation of inflammatory processes, oxidative stress, remodeling of cellular function and morphology, and disturbance of metabolic pathways. The growing interest in the characterization of protein post-translational modifications and the importance of handling large datasets using a systems biology approach are also discussed.

## 1. Introduction

DN is an endemic complication of diabetes and the first cause of ESRD worldwide. The contributing causes of DN pathogenesis and progression are still poorly understood but chronic hyperglycemia and high blood pressure represent the main risk factors for disease onset.

*Hemodynamic and Biochemical Background*. In the early stages of DN, high systemic blood pressure usually determines an increase of the intraglomerular pressure and glomerular filtration rate (GFR) which results in glomerular hyperfiltration [1]. From the biochemical point of view, hyperglycemia* per se* sustains the accumulation of advanced glycation end products (AGEs), altering the electronegativity of the cell; additionally AGEs bind proteins of the extracellular matrix (ECM) inhibiting their degradation. AGEs accumulation can induce an increased production of reactive oxygen species (ROS) and a transcriptional activation of different proinflammatory and profibrotic molecules, including TGF-beta [2, 3]. The high glucose-mediated induction of TGF-beta and the central role of this growth factor in DN progression represent the few defining constants in the pathogenesis of DN [4].

*Clinical and Histological Hallmarks of DN*. The earliest clinical signs of DN include a slight but persistent urinary excretion of albumin (microalbuminuria) and a temporary increase of the glomerular filtration rate (GFR). These clinical signs, along with the presence of hyperglycemia, are often considered sufficient indicators of DN [5, 6]. Today, extensive evidence shows that DN is not the only type of renal damage that can be found in diabetic patients [7, 8] and kidney biopsy, although highly invasive, remains the diagnostic gold standard. The histological hallmarks of DN include hyperproliferation of the mesangial cells, thickening of the glomerular basement membrane (GBM), podocyte effacement, tubulointerstitial fibrosis, and nodular accumulations of ECM (Kimmelstiel-Wilson lesions) in the glomerulus [9].

Given the high prevalence of type 2 diabetes (T2D) and the diagnostic limitations currently associated with kidney biopsy, there is an impending need for new, accurate, and easily accessible biomarkers of disease.

In this review we will try to outline a system biology overview on DN by recapitulating the main annotations obtained at different levels of molecular investigation. Only those studies investigating human samples will be described; the murine models of DN in fact, although undergoing albuminuria, mesangial expansion, and podocyte loss, do not develop severe glomerulosclerosis and tubulointerstitial fibrosis [10]. Also, as substantial differences exist in the etiology and prevalence of type 1 and type 2 DN, the articles discussed in this paper apply to DN secondary to type 2 diabetes (T2DN). As an exception, works describing biomarkers of kidney damage in T1D that have been further validated in T2DM and* vice versa* and those reporting potential prognostic biomarkers, because of their particular importance in predicting the progression of renal damage, have been also discussed in the present work. All the annotations discussed in this review are also listed in Tables 1, 2, 3, 4, and 5, categorized according to whether they summarize the genetic and transcriptomic signature of coding or noncoding RNA molecules and the epigenetic proteomic and metabolomic markers, respectively.

## 2. Genetic Profiling of DN

Genetic variation is present under different forms in the human genome, ranging from single nucleotide polymorphisms (SNPs) to large, structural, chromosomal rearrangements. Today we know that genetic variation infers disease susceptibility and collective effort aims at identifying the precise loci for DN susceptibility. Different methodological strategies can be used to characterize the genetic risk for a disease, either targeted or genome-wide, according to whether* a priori* hypothesis of the candidate regions for disease susceptibility exists. In genome-wide association studies (GWAS), for instance, the whole genome is screened for new, previously uncharacterized single nucleotide polymorphisms (SNPs).

Prior to the development of the modern high-throughput technologies such as chip-based microarray analysis and next-generation sequencing, the inheritance of disease susceptibility was investigated through genetic linkage in families. Basically, individuals within the same families were sequenced for a collection of genetic SNPs in order to identify those SNPs segregating with the disease. This approach led to the identification of many variants responsible for disease susceptibility but it proved mostly suitable for the study of single gene disorders. For complex, common complications like T2D in fact, progression is very likely driven by multiple alleles simultaneously, each having a small correlation to disease progression if inherited individually. This implies that a big population needs to be genotyped in order to detect the common variants responsible for the increased genetic risk.

In the field of DN, there is extensive evidence for genetic contribution to disease susceptibility. In 1989, Seaquist et al. showed that diabetic siblings of patients with DN were more at risk for developing DN compared to diabetic siblings of diabetic patients without proteinuria [99]; epidemiologic studies also indicate that the prevalence of DN varies among ethnic groups [100]. These observations, along with the consideration that only a subset of patients with diabetes develops DN, drove the search for the genetic determinants of DN susceptibility.

One of the most consistent annotations in the field is probably the genetic variation on chromosome 18. In 2002, a family-based linkage analysis performed in T2DN Turkish families and affected sibling pairs of Pima Indians reported a strong evidence for the localization of a DN susceptibility locus mapping to chromosome 18q22.3-23 [12]. Researchers were not able to pinpoint the precise susceptibility gene but the same locus was also detected in a T2DN African American population [11]. Later studies on chromosome 18 led to the identification of a susceptibility marker within the carnosine dipeptidase 1 (CNDP1) gene, and it was also described how the shortest allelic form of the CNDP1 gene was more common in the absence of nephropathy [15]. The CNDP1 gene encodes the secreted enzyme serum carnosinase that degrades carnosine, a protein controlling the formation of AGE molecules [101]. As previously discussed, AGE's accumulation is a phenotypic sign of DN. Similar results were obtained in a meta-analysis study when investigating a multiethnic population with T2D-ESRD [16]; a recently published meta-analysis confirmed the association of the carnosinase D18S880 microsatellite polymorphism with DN susceptibility in a T2D Caucasian population although no significant association with T1DN could be found [17].

In a very recent candidate-gene driven study, Palmer et al. performed a genotyping of several SNPs across 22 DN candidate genes in a large cohort of African Americans with T2D and ESRD. After adjustment for the APOL1 G1/G2 alleles, known to be associated with nondiabetic ESRD in this population, the most significant signals were observed downstream of the CNDP1 gene, at chimerin 2 (CHN2) locus and within angiotensin II receptor type 1 (AGTR1) gene [13]. In another work, to investigate the impact of oxidative stress on disease initiation, the polymorphic variants of 7 genes involved in the antioxidant defense were evaluated: SOD2, p22 phox, CAT, MPO, GSTP1, GSTT1, and GSTM1. Despite the commonly recognized link between oxidative stress and diabetes, authors claim that no association could be found in Caucasian T2D patients [102].

In one of the first DN genome-wide genotyping studies, authors reported the engulfment and cell motility 1 (ELMO1) gene on chromosome 7p as a likely candidate for disease susceptibility in a Japanese patients cohort with T2D [19]. In a cellular system engineered to overexpress ELMO1, they furthermore observed increased expression of extracellular matrix (ECM) protein genes and decreased expression of matrix metalloproteinases [103]. The same susceptibility locus was also identified in a T1DN Caucasian cohort [18]. Finally, recent data from a meta-analysis study suggests the ELMO1 association with DN exclusively in the T2D Asian subgroup [16].

In a population of Pima Indians with T2D, the GWAS of over 100,000 SNPs led to the identification of several loci with significant association for ESRD susceptibility, with the strongest signal located in the intronic region of the of PVT1 gene [22]. Some of these findings were also replicated in an ethnically different population with T1D [23].

In a GWAS performed on a large cohort of African Americans with T2D and ESRD, five gene regions with evidence of association with DN were detected, nominally, SASH1, RPS12, AUH, MSRB3-HMGA2, and LIMK2-SFI1. Some of these SNPs however were later found to contribute to all-cause ESRD [14].

In order to establish a comprehensive, well-defined DNA biobank for the genotyping of DN in T1D in particular, the Genetics of Kidneys in Diabetes (GoKinD) study was undertaken [104]. The first results of this genome-wide scan were reported by Pezzolesi et al. in 2009. Authors claimed that although no SNP achieved genome-wide significance, strong association was found near the 4.1 protein ezrin, radixin, and moesin [FERM] domain containing 3 (FRMD3) locus and near the cysteinyl-tRNA synthetase (CARS) locus [105]. Further studies confirmed the 9q21.32 region (upstream of FRMD3) as a susceptibility locus for T2DN in several unrelated study populations [20, 21].

Despite all the effort currently invested into this field of research, at present it is still impossible to predict those diabetic patients with a higher risk for developing DN. Indeed, in almost all the studies published so far on DN susceptibility, diagnosis was based almost exclusively on the presence of hyperglycemia and proteinuria; therefore, it is not possible to exclude that the inconsistencies among the findings could be linked to a misclassification of the renal damage in the diabetic population.

The genetic markers cited in this paper are also summarized in Table 1.

## 3. Transcriptome Profiling of DN

The transcriptome represents the part of genome that is transcribed and includes both coding and noncoding RNA molecules. When studying the transcriptome, as for genetic studies, either targeted or genome-wide approaches can be used. RNA-sequencing (RNA-seq), arrays, and quantitative PCR (qPCR) are the techniques employed routinely to assess RNA expression. qPCR is very sensitive and even subtle changes can be detected precisely; arrays on the other hand are very high-throughput but also less sensitive. RNA-seq takes advantage of the recent next-generation sequencing platforms and it has rapidly become the method of choice for transcriptome profiling. The main advantages of RNA-Seq are its very high resolution (down to a single nucleotide), its potential to detect novel transcripts, its ability to measure either primary transcripts or spliced mature mRNAs. Given the plethora of gene expression data available in the literature, only the research on DN kidney tissue or urine will be discussed. All the coding and noncoding RNA markers cited in this paper are also summarized in Tables 2 and 3, respectively.

### 3.1. Coding RNA Studies

The first transcriptomic signature of DN kidney was published in 2004. Using an array-based approach, Baelde et al. assayed the glomerular gene expression profile of T2DN and morphologically normal, nondiabetic kidneys. The results of this genome-wide analysis indicated that 96 genes were upregulated in T2DN, including aquaporin 1 (AQP1), calpain 3 (CAPN3), hyaluronoglucosidase, and platelet/endothelial cell adhesion molecule (PECAM-1). Over 500 genes were downregulated, including bone morphogenetic protein 2 (BMP2), vascular endothelial growth factor (VEGF), fibroblast growth factor 1 (IGF-1), insulin-like growth factor binding protein 2 (IGFBP-2), and nephrin. In the same manuscript, authors confirmed reduced expression of VEGF and nephrin in renal biopsy specimens from additional DN patients at both the protein and RNA levels [32].

To explain the existing inconsistencies between human and murine progressive DN, microdissected biopsies from controls, early and progressive T2DN patients underwent global gene expression profiling through microarray hybridization. Preliminary results, later confirmed using qPCR, revealed an upregulation of Jak-2 and a compromised expression of several members within the Jak/Stat signaling pathway which could not be detected in either* db/db* C57BLKS or diabetic STZ-treated DBA/2J mice [50].

More recently, Woroniecka et al. performed the transcriptome analysis on microdissected kidney biopsies from DN patients, healthy living transplant donors, and patients undergoing tumor nephrectomies (analyzing the histologically normal kidney tissue). The microarray-derived expression profiles indicated that several podocyte-specific transcripts were downregulated, including PLCE1, PTGDS, NPHS1, NPHS2, SYNPO, PLA2R1, WT1, CLIC5, and PODXL. Glomerular transcripts showing upregulation included IGH, C3, COL1A2, CXCL6, and COL6A3. In the tubular compartment instead, authors detected increased expression of different transcripts including IGH, IGL, COL1A2, and COL3A1 [37].

Several reports analyzed the gene expression of both the glomerular and tubular compartments of T2DN kidney biopsies. Among the mRNA transcripts detected as enriched in the glomerular compartment of T2DN individuals are MRP8 [52], WNT1, WNT2B, WNT4, WNT6, WNT16, DKK3, and Lef1 [42], PKC*α* [58], FSP1 [44], ANGPTL2 [30], and ACE [26]. Decreased expression for ACE2 [26], VEGF [64, 106], CTGF, nephrin, podocin, and WT1 [41] was also reported in T2DN glomeruli.

When assaying microdissected, tubule-rich renal biopsies from patients with T2DN, IHG-1 [48], IL6, CCL2 CD68, and CCR5 [38] were increased, while TLR4 was overexpressed in both glomeruli and tubules of microalbuminuric and overt DN [38].

Using biopsy material collected by the European Renal cDNA Bank, the gene expression of tubulointerstitial mRNA from human DN kidneys was compared to that of living donors, cadaveric donors, and patients with minimal change disease through a combined microarray profiling and qPCR validation approach. Results indicated dysregulation of specific NF-*κ*B targets, highlighting the existence of an inflammatory signature characteristic of progressive DN. Eight genes in particular were induced in T1DN and T2DN relative to controls: CCL5/RANTES, CXCL10/IP10, EDN1, VCAM1, HLA-A, HLA-B, IFNB1, and B2M [35].

Further work performed using the European Renal cDNA Bank material highlighted additional mRNA transcripts as dysregulated in T2DN kidney when compared to normal tissue. Within the glomerular compartment in particular, NRP1 and NRP2 were significantly lower in T2DN [55], while SMPDL3b was increased [61]. Within the tubulointerstitial compartment, upregulation of MMP7 [51] and FGF-2 [43], of the unfolded protein response genes HSPA5, HYOU1, and XBP1 [47] and of the apoptosis-related genes TRAIL and OPG [56], were observed.

In other cases, the expression of several transcripts was assessed on whole T2DN kidney tissue.

Upregulated mRNAs included HDAC2, HDAC4, and HDAC5 [34], B7-1 [36], Stat1 [33], TNFAIP8 and TIPE2 [62], PRKC-beta [59], VEGF [65], UII and UT [63], PDGF-A and PDGF-B [57], LOX1, LDLR, and CD36 [24], Jagged1/Hes1 [46], and Gremlin [45, 46].

Decreased transcription was detected for autophagy-related genes Beclin 1, LC3 [33, 34] and ATG7 [34], CXCL16, ABCA1, ABCG1, and apoE [24], Timp3, FoxO1 and FoxO3A, Atg5, and Atg8 [33], ANKRD56 and ENTPD8 [31], and nephrin [54].

In other works the study design was developed to compare T2DN with other glomerulopathies. Using a qPCR based approach, the tubulointerstitial compartment isolated from kidney biopsies of both DN patients, living donors, and minimal change disease patients was profiled specifically for the expression of 202 candidate genes involved in molecular pathways contributing to DN progression. Results showed a decreased expression of VEGF and EGF, while Collagens I and IV, fibronectin 1, and vimentin as well as matrix metalloproteinases 2, 7, and 14 and tissue inhibitor of metalloproteinases 1 and 3 were increased [39]. In another study, increased IRS2 mRNA was detected in DN patients compared to controls, while no significant changes IRS2 expression were present in biopsies from patients with focal-segmental glomerulosclerosis or membranous nephropathy [49].

Low expression of ROBO2 mRNA was present in DN compared to nephrosclerosis, focal-segmental glomerulosclerosis, membranous nephropathy, and control pretransplant biopsies [60].

A strong specific induction of COL8A1 and COL8A2 mRNAs expression was found in both glomerular and tubular compartments of biopsies from patients with T2DN versus control pretransplant biopsies, benign nephrosclerosis, and focal-segmental glomerulosclerosis [40]. Finally, increased ACE expression was observed in T2DN biopsies compared to benign nephrosclerosis, minimal change nephrotic syndrome, and lupus nephritis [25].

Aiming to develop a diagnostic tool for early DN diagnosis, Zheng et al. designed a PCR-array platform to detect expression changes in 88 genes simultaneously and employed it in a pilot study where the urinary sediment of DN patients was assayed. Authors found that several mRNAs were significantly increased in DN compared to healthy controls, in particular, NOTCH3, ACTN4, CDH2, ACE, FAT1, COL4A1, SYNPO, and TWIST1 [27]. Similar studies investigated the mRNA derived from the urinary sediment of T2DN patients. Increased mRNA levels of podocalyxin, CD2-AP [29], nephrin, WT-1 [28], *α*-actinin 4 podocin, and synaptopodin [28, 29] were found in the DN group compared with controls. Finally, in another work, authors claim that urinary expression of nephrin and podocin was useful for distinguishing diagnostic groups (IgA nephropathy, minimal change disease, and membranous nephropathy) as well as predicting renal function decline [53].

### 3.2. Noncoding RNA Studies

Until a few years ago, the molecular profiling of DN was mainly focused on the characterization of mRNA transcripts. Over the last decade however, much interest has converged toward the profiling of noncoding RNA (ncRNA) molecules. The ability of ncRNAs to modulate gene expression along with the discovery that they can be detected in biofluids and are fairly stable makes them ideal biomarker candidates.

microRNAs (miRNAs) are probably the most studied ncRNAs; they are short, single-stranded, highly conserved, and tissue-specific. miRNAs regulate protein synthesis through perfect partial match binding to their precursor messenger RNA. The partial match binding feature allows miRNAs to bind hundreds of targets simultaneously; accordingly the dysregulation of even one single miRNA molecule can profoundly influence the gene expression profile of the surrounding environment. For a complete review on miRNAs biogenesis and function refer to [107, 108]. In the field of DN, the majority of miRNA's profiling studies was performed on cellular and animal models. More recently, with the surprising discovery that miRNAs can be released and carried into the extracellular environment, different body fluids are being characterized in their miRNA's content.

The first miRNA to be recognized as relevant contributor to DN progression was miR-192 [109]. Initially identified in a mice model of DN, miR-192, along with miR-377, miR-337, and miR-129, was later discovered as being enriched in human mesangial cells (MCs) exposed to high glucose [67]. Interestingly, when assessing miR-192 in human DN kidney, expression levels not only are reduced but also inversely correlate with severity of kidney disease [72], raising once again the issue about the appropriateness of the currently available animal models for DN.

miR-21 has recently emerged as a marker for fibrosis in many complications [110, 111]; unsurprisingly, increased miR-21 expression was also detected in human T2DN kidney biopsies relative to healthy controls [74].

Except for the previously mentioned DN kidney profiling from Krupa et al., the array-based miRNome analysis of T2DN kidneys was recently published by Huang et al. and uncovered miR-155 and miR-146a enrichment in these samples [69]. These two are the only works describing the miRNome of human DN kidney; noteworthy, the existence of strict renal biopsy policies in most nephrology clinics might be a limiting factor in terms of sample collection and availability. In parallel, the urgent need for novel biomarkers of diagnosis and progression shifted priority to the profiling of more accessible samples, such as biological fluids.

Using a qPCR based approach, Argyropoulos et al. were the first to perform the urinary miRNA profiling of T1D patients with and without proteinuria. Results showed that miR-323b-5p, miR-221-3p, miR-524-5p, and miR-188-3p were underexpressed in albuminuric relative to nonalbuminuric patients, while miR-214-3p, miR-92b-5p, hsa-miR-765, hsa-miR-429, miR-373-5p, miR-1913, and miR-638 were overexpressed [71]. In a similar study performed on the RNA content of urinary exosomes, authors showed that miR-130a and miR-145 were enriched in T1D patients with microalbuminuria compared to normoalbuminuric subjects, while miR-155 and miR-424 were reduced [68].

In a work aimed to determine the urinary levels of all miR-29 family members (miR-29a, miR-29b, and miR-29c), miR-29a was significantly increased in albuminuric T2DN patients compared to normoalbuminuric patients and it also correlated with the degree of albuminuria [75].

In the work from Szeto et al., when comparing the urinary sediment of patients with either IgA nephropathy, DN, or hypertensive nephrosclerosis, miR-15 was decreased in DN samples compared to other groups [70]. Similarly, in another work authors found that miR-192 levels were reduced in urinary sediment of DN patients compared to both healthy controls and patients with either minimal change nephropathy, focal glomerulosclerosis, membranous nephropathy, or other diagnosis groups [73].

miRNAs expression was also measured in venous blood from T2D Han Chinese patients with and without albuminuria. Using a microarray-based approach, authors identified several differentially expressed miRNAs in the different study population and confirmed miRNA let-7a downregulation using qPCR. Very interestingly, authors also observed how the distribution of a specific variant within let-7a (rs1143770) was significantly higher in diabetic patients (with and without albuminuria) relative to control subjects [76].

Finally, dysregulation of a new class of noncoding RNA molecules has emerged as being potentially involved in different complications, including kidney disease. Among these noncoding RNA molecules, recent effort aims to characterize the so-called long noncoding RNAs (lncRNAs). Compared to miRNAs lncRNAs are longer than 200 nucleotides and are poorly conserved. This led to the initial assumption that lncRNAs were not biologically relevant. Today we know that lncRNAs contain individual domains and structural motifs that allow them to specifically associate with DNA, RNA, and/or protein and thus regulate their function.

The first lncRNA identified in kidney disease was PVT1. As previously discussed, multiple experimental evidence, from different ethnic populations, suggested a link between diabetic kidney disease and genetic variants within the PVT1 locus [22, 23]. PVT1, whose increase is significant in mesangial cells stimulated with high glucose, can induce the expression of plasminogen activator inhibitor 1 (PAI-1) and transforming growth factor beta 1 (TGF-*β*1) [112]. Noteworthy, six different miRNAs are encoded within the PVT1 gene; therefore, authors investigated whether an alteration in PAI-1 and TGF-*β*1 gene expression was ascribable to the PVT1 lncRNA transcript itself or whether it was the result of a mutation within the miRNAs encoded in the PVT1 gene. Results showed that both PVT1 lncRNA and miR-1207-5p were induced by high glucose independently and they both contributed to ECM accumulation in the kidney [66].

## 4. Epigenetic Studies in DN

The term epigenetics refers to all those dynamic structural changes that, while not resulting from an alteration in the DNA sequence, affect gene expression and can be inherited. Epigenetic modifications, such as DNA methylation, histone methylation, and histone acetylation, modify the accessibility of the chromatin and thus modulate transcription. They are responsible for the phenotypic differences within cell types and explain why the gene expression profile of an organism can change so profoundly during development. Unlike genetics, epigenetics is highly susceptible to influences from the environment; therefore, the understanding of its regulatory machinery offers an incredible opportunity for disease management.

The study of epigenetics in diabetic kidney disease is still in its embryonic phase although increasing evidence indicates metabolic memory as a consequence of long-lasting epigenetic modifications contributing to DN progression [113]. In 2007 Geisel et al. analyzed the promoter methylation of the stress response protein p66Shc, previously shown to increase susceptibility to oxidative stress and atherosclerosis [114]. In peripheral blood mononuclear cells isolated from ESRD patients and control subjects, authors demonstrated that increased p66Shc expression in ESRD group was linked to a significant reduction in the methylation of its promoter region [77].

Using an array based approach, the genome-wide promoter DNA methylation of 192 T1D patients was analyzed searching for any possible association with DN. The analysis was conducted using DNA extracted from peripheral blood cells as these include the T cell population responsible for islet beta cells destruction in T1D. Importantly, among the several CpG islands showing correlation with DN development, results uncovered one in particular (rs10081672), located upstream of the UNC13B gene. Additionally, this region is in strong linkage disequilibrium with rs13293564, a variant associated with DN susceptibility. Importantly, depending on which allele is present in rs10081672, a CpG site is either created or abrogated, thereby affecting transcription factor binding [78].

In another work, the genome-wide DNA methylation of diabetic patients with ESRD and diabetic patients without nephropathy was compared with the aim to identify novel disease biomarkers for noninvasive diagnosis. Patients' saliva was employed as starting material for DNA extraction while the study population included African Americans and Hispanic individuals. Results highlighted differential methylation at two or more CpG sites in 187 genes between the two groups. Interestingly, many of these genes are involved in inflammation, oxidative stress, ubiquitination, fibrosis, and drug metabolism, and some in particular are even known for their genetic association with DN, suggesting once again a very close connection between genetic dysregulation and epigenetic dysregulation in the pathogenesis of DN [115].

A recent paper from Hasegawa et al. demonstrated that Sirt1, a protein deacetylase that targets histones and transcription factors, is reduced in STZ-treated mice. Using a transgenic mouse model authors also elucidated the interaction between Sirt1 expression and CpG methylation of Cldn1, a gene encoding for the protein Claudin-1. Claudin-1 is a tight junction protein involved in cell-to-cell adhesion and authors suggest that its epigenetic-mediated induction is responsible for podocyte effacement and proteinuria. In support of this hypothesis authors also revealed the correlation between proteinuria and Sirt1 expression in human DN kidney [116].

Finally, Reddy et al. elegantly demonstrated the link between the protective effect of angiotensin II receptor antagonist, losartan, and its ability to reverse specific epigenetic modifications in the glomeruli of diabetic* db/db* mice [117]. The epigenetic marks cited in this paper are listed in Table 4.

All these experimental evidences show that epigenetics holds the potential to allow a temporary and reversible manipulation of the gene expression, conferring protection from disease progression. They also highlight the importance of understanding the epigenetic contribution to DN progression.

## 5. Proteomics Studies in DN

The proteome probably represents the most complete expression of the potentialities of a living organism since it focuses on the set of proteins, expressed by the genome, that regulate biological and metabolic cell function. The “proteomics,” formally defined as the massive and mass spectrometric-based analysis of the proteome, is a complex and interdisciplinary matter requiring expertise spanning from chemistry to biology and bioinformatics, in order to reveal the meaning of complex protein datasets of a biological sample in physiological and pathological conditions. Unlike genomics studies, based on the analysis of biological samples that may be expanded artificially making complex studies from little starting material possible, proteomics requires a larger amount of starting sample that can be easily available in biological fluids rather than in the tissues or cells. For this reason, proteomic studies in nephrology are more oriented to the analysis of biological fluids and have led, in the last decade, to the identification of a number of putative biomarkers that are expected to enter shortly into the clinical practice [118].

In the next paragraphs we will discuss the main application of proteomics to the identification of new potential biomarkers of DN in kidney tissues and biological fluids with a special emphasis on the new emerging potentialities of the post-translational modifications (PTMs) screenings. The proteomic markers discussed in this paper are also reported in Table 5.

### 5.1. Kidney Tissue

Glomerular damage plays a critical role in the onset of DN making this renal compartment a key target for proteomic investigation [119]. However, only few proteomic studies have been carried out on isolated glomeruli since, in general, renal biopsy is rarely carried out on diabetics patients and the number of isolated glomeruli, when starting form biopsy material, is too scarce to produce homogeneous preparations of individual specimens and to extract adequate glomerular protein amounts for deep proteomic studies. Recent methodological improvements [120] have now permitted the extraction of intact and unmodified proteins from formalin fixed paraffin embedded (FFPE) samples thus making available the use of vast archive of kidney tissues for proteomic analysis. Proteomic analysis of isolated glomeruli, obtained by Laser Capture Microdissection (LCM) [121], allowed the identification of over 100 differentially expressed tissue proteins between DN and nondiabetic glomeruli [79]. Notably, the results of this study probably underestimates the differences of the glomerular proteome since it was carried out on FFPE tissues derived from autopsy cases undergoing postmortem proteolysis [122]. However, among differently expressed proteins, nephronectin, a protein implicated in the assembly of extracellular matrix and nephrogenesis [123], was confirmed as differently expressed in DN tissue specimens using immunohistochemistry. A similar study reported increased expression of C3 and the membrane attack complex (C5b-9) and a marked reduction of podocyte-associated proteins and antioxidant proteins in DN [80]. Even if these proof of concept studies demonstrate the usefulness of FFPE tissue proteomics, the potentialities of this approach are still prevented by the poor availability of tissue specimens that limits the identification of the key molecular events involved in the onset and progression of DN.

### 5.2. Biofluids

Biofluids encompass any liquid originating from inside the bodies of living organism. Among the body fluids proteomics has been mostly applied to urine and serum/plasma. Rossing and colleagues reported, in urine of T1D patients with DN, a panel of 65 urine biomarkers, mainly composed of collagen fragments, that was further validated in a multicentre independent cohort of T2DM patients [124, 125]. Zürbig et al. expanded the 65 peptides classifier to 273 and demonstrated its ability to predict the occurrence of the microalbuminuria in T1D and T2DM normoalbuminuric patients [126, 127]. These data were recently confirmed in another independent study that specifically identified subsets of urine biomarkers able to predict to the transition from normo- to microalbuminuria or from micro- to macroalbuminuria [81] indicating that the appearance of collagen fragments in urine of T2DM patients may have both diagnostic and prognostic values. Potential predictive biomarkers have been also described in urine samples of T1 diabetic patients [82]. LC/MS/MS analysis of 22 T1D normoalbuminuric patients developing microalbuminuria after 6 years median follow-up allowed identifying a set of potential predictive biomarkers that were further validated by ELISA assay. Of note, the introduction of these proteomic biomarkers (THP, progranulin, alpha-1-glycoprotein, and clusterin) into the baseline model that included diabetes duration, baseline Albumin Excretion rate (AER), HbA1c, cystatin C, and uric acid improved the prediction of renal function worsening from 84% to 89%.

Jin et al. used* Isobaric Tags for Relative and Absolute Quantification* (iTRAQ) and LC/MS/MS to quantify and identify a set of urinary proteins differentially excreted between normoalbuminuric and microalbuminuric T2DM patients. Three protein biomarkers, namely, alpha-1-antitrypsin, alpha-1-acid glycoprotein 1, and prostate stem cell antigen, were included in a multiplex assay that was able to correctly classify normoalbuminuric and microalbuminuric T2DM patients with about 92% accuracy [83].

Dihazi et al. identified and validated, by SELDI-TOF/MS, two mass peaks corresponding to B2-microglobulin and ubiquitin ribosomal fusion protein that were selectively and differently excreted in nephropathic diabetic patients [84]. We further refined this study by selecting only diabetic patients with biopsy-proven Kimmelstiel-Wilson lesions and identifying both urinary B2-microglobulin and free ubiquitin as specific biomarkers of diabetic glomerulosclerosis over other nondiabetic kidney lesions [85]. Although the overall analysis of the urine proteome is up to now the most used way to search for disease-specific biomarkers, the future of this matter will be the analysis of well-purified proteins subfractions since it may provide more detailed information about simplified proteomes and potentially improve the knowledge of specific pathways. Until few years ago, the most useful way to reduce the proteome complexity was the selective antibody-based depletion of the most abundant proteins. In the last few years, the enrichment of post-translationally modified proteins has begun a new strategy to highlight functionally interesting proteins. Two emerging branches in this context are phosphoproteomics and glycoproteomics. Protein phosphorylation is a key player in the regulation of most cell pathways; thus, phosphoproteome screening of urine samples may represent a precious source of information about deregulated cell processes in many kidney diseases including DN. However, up to now, urine phosphoproteome analysis has not been applied yet to soluble proteins in DN and other CKD probably because most of the historical collections of urine samples have not been prepared and stored in presence of phosphatases inhibitors that, preventing the liability of this PTMs, may ensure more reproducible results. On the contrary, the analysis of the microvesicular fraction (i.e., exosomes) that originates from renal epithelial cells and are released into urine may be, at the moment, more useful to study this kind of PTM as the presence of the exosomes' membrane may preserve PTMs by protecting their protein content from spontaneous degradation and dephosphorylation by proteases or phosphatases, respectively [128]. Zubiri et al. have already published the first proteomic study on urine exosomes of DN patients demonstrating the potentiality of this microvesicular screening for identifying DN specific biomarkers [86]. Specifically, 3 over the 25 most significant differently expressed proteins, namely, voltage-dependent anion-selective channel protein 1 (VDAC1), Isoform 1 of histone-lysine N-methyltransferase MLL3, and alpha-1-microglobulin/bikunin precursor (AMB), were also validated. Of note, MLL3, a specific tag for epigenetic transcriptional activation, was detected only in DN exosomes, thus emphasizing the potential importance of epigenetic mechanisms in the pathophysiology of DN. Furthermore, Gonzales et al. have recently reported the first phosphoproteomic screening of the urine exosomes in healthy subjects [129]. It is reasonable to think about the forthcoming application of the exosomes' phosphoproteomics as a new way to identify specific deregulated patterns in kidney diseases. As for phosphoproteomics also glycoproteomics of urine samples is in its infancy. At the moment, only one paper has applied this approach to the study of CKD identifying a number of urinary proteins involved in immune/stress response and many biological functions like homeostasis, platelet degranulation and coagulation, transport, and secretion [130]. Due to the importance of the glycoproteomics in cell-cell interaction and signalling cascades, it is reasonable that many further studies will be planned in the next year to understand, by screening this specific subset of proteins, the molecular mechanisms involved in damage progression of specific nephropathies including DN. Interestingly, the usefulness of the glycoproteomics for the diagnosis of DN has been recently reported in plasma where thirteen significantly upregulated glycoproteins were described in DN patients compared to T2DM patients without nephropathy [87]. Among these, increased plasma levels of glycated lumican, vasorin, and retinol binding protein-4 were validated by immunoblotting and showed potential specificity for DN. By using a different proteomic strategy, Kim and coworkers reported that increased plasma levels of glycated PEDF, apolipoprotein J precursor, hemopexin, immunoglobulin mu heavy chain, and immunoglobulin kappa chain correlated with poor glycaemic control in T2DM patients while glycated prekallikrein and complement factor C4B3 correlated with microalbuminuria and other glycated proteins such as hemopexin precursor, serine proteinase inhibitor, alpha-1-antitrypsin, and haptoglobin-related protein were associated with DN [88]. These studies confirmed the potentiality of the plasma glycoproteome for the identification of reliable biomarkers of DN and their importance is emphasized by the consideration that the overall analysis of serum/plasma proteome is challenging because the candidate biomarkers are generally present in trace amounts. Of note, there is an alternative way to reduce the complexity of this biological fluid, namely, the prefractionation of the samples, achieved by several known strategies before the analysis [131], that allow removing the large background of nonrelevant and abundant proteins and may favour the discovery of potential candidate biomarkers. Up to now only few studies have used this approach to analyse the serum [89, 132] or plasma [133] proteome of T2DM patients. These studies have reported extracellular glutathione peroxidase (eGPx) and apolipoprotein (ApoE) as potential diagnostic biomarkers of DN and vitamin D-binding protein (DBP) as early biomarker of renal damage in T2DM. Overall many independent studies are showing an increasing number of new biomarkers that are potentially useful for both early diagnosis and monitoring of the disease and to understand ever more deeply its pathogenesis.

## 6. Metabolomics Studies on DN

Metabolomics is a systematic evaluation of small molecules (i.e., metabolites) that may provide fundamental biochemical insights into disease pathways, drug toxicity, and gene function. Metabolomics profiling is generally carried out by Nuclear Magnetic Resonance (NMR) and MS-based profiling each with advantage and limitations [134]. Two main strategies may be adopted for metabolomics analysis of biological samples: targeted and untargeted profiling. The targeted profiling focuses only on sets of few metabolites generally included in specific metabolic pathways while untargeted analysis provides a comprehensive evaluation of the metabolome without any* a priori* hypothesis on the metabolic pathways. Targeted analysis is an essential tool for the investigation of biological mechanisms rather than for biomarkers discovery; in fact it is a quantitative approach that allows quantification of each metabolite of an interested metabolic pathway through the use of isotope-labelled standards [135]. Untargeted approach is instead more suitable for biomarker discovery since the whole metabolic profile of cases and controls may allow identification of disease-correlated biomarkers. As obvious, the latter approach needs, as for proteomics, further data analysis through supervised statistical methods in order to construct disease-specific metabolomics classifier further sequenced by mass spectrometry. In the last years, the optimization of the separation techniques has allowed the selectively purification of specific class of metabolites such as phospholipids and fatty acids, leading to the development of new more focused untargeted analysis such as “phospholipidomics.” As for proteomics, most of the metabolomics studies have been carried out on biofluids, namely, urine and serum/plasma. All metabolomic markers cited in this paper are also reported in Table 6.

### 6.1. Urine Metabolomics

Urine metabolomics may offer direct insights into biochemical pathways linked to kidney dysfunction since a variety of metabolites are concentrated by the kidney and excreted in urine. Sharma et al. [90] used targeted analysis to investigate the urinary excretion of 94 metabolites in healthy subjects (HS) and T2DM patients with (DM+CKD) or without (DM-CKD) CKD. Thirteen metabolites differently excreted between T2DM patients and HS were also useful to differentiate DM+CKD from DM-CKD. Interestingly, 5 out 13 metabolites were differently excreted between DN and other CKD, thus being specifically associated with the diabetic kidney disease while 8/13 reflected metabolic changes shared by diabetic and nondiabetic CKD. Most of the less excreted metabolites in DN group were water soluble organic anions and functional analysis correlated them with impaired mitochondrial function in DN. Very recently, Pena and colleagues carried out an untargeted analysis of urine and plasma metabolome by GC-MS and reported the possible usefulness of a set of metabolites to predict the development of DN on top of the traditional renal risk markers, namely, baseline urinary albumin excretion and baseline estimated glomerular filtration rate [91]. In this prospective study, 24 normo- to microalbuminuria case/controls pairs and 21 micro- to macroalbuminuria case/controls pairs were enrolled. The metabolomic profiles of micro- to macroalbuminuria case/control pairs show significant differences while normo- to microalbuminuria pairs remained unchanged. Specifically they reported two plasma metabolites (butenoylcarnitine and histidine) and three urine metabolites (hexose, glutamine, and tyrosine) significantly differentially excreted in microalbuminuric patients prone to develop macroalbuminuria. The area under receiving operating characteristic (ROC) curve arising from the integration of these urine and plasma metabolites to a reference model based on baseline eGFR and urine albumin excretion passed form 84% to 99% correct prediction. Although these results appear impressive, as the authors suggest, they still need to be managed with care until a validation study on larger and independent cohorts will be set up. Some of the identified metabolites may have direct link with the pathopysiology of diabetes and its chronic complications since, for example, butenoylcarnitine plasma accumulation has been related to the excessive yet incomplete mitochondrial oxidation of fatty acids [136], possibly attributable to a lower mitochondrial number and reduced oxidation capacity in T2D tissues [137] while histidine, a modulator of inflammation and oxidative stress, may be correlated with impaired inflammation and oxidative stress in T2DM and CKD patients. It is worth noting that both studies stressed the importance of mitochondria dysregulation in the pathogenesis of DN. Urine metabolomics has been also applied to type 1 diabetic patients in order to identify predictive biomarkers of renal function worsening [92]. Metabolite profile of baseline 24 h urine samples of 52 type 1 diabetic patients (26 stable normoalbuminuric and 26 progressed toward microalbuminuria in 5.5 years' follow-up) was carried out by LC/MS and GC-MS. Multivariate logistic regression analysis of GC-MS and LC/MS dataset showed 65% and 75% predictive power after cross-validation, respectively. Twenty-one and 14 compounds showed a significant contribution to the logistic regression model based on GC-MS and LC/MS dataset, respectively. Most of the identified GC-MS compounds were carboxylic compounds, acidic metabolites, and endogenous amino acids not showing a documented direct relation to DN while LC-MS dataset reveals specific compounds related to impaired fatty acids metabolism, detoxification system, and gut microbiome.

### 6.2. Serum and Plasma Metabolomics

Serum and plasma metabolomics has been carried out of both whole samples and specific subfractions. Marrachelli and coworkers [93] performed both genomic and metabolomic screening of over 1500 Caucasian T2DM patients, characterized the serum metabolome profile of the microalbuminuric patients by Nuclear Magnetic Resonance (NMR), and correlated it with specific genotypes, thus reporting a potential predictive value of the genotype on the onset of microalbuminuria in T2DM. Furthermore, Hirayama et al. [94] reported, in T2DM patients, 19 serum metabolites including creatinine, aspartic acid, *γ*-butyrobetaine, citrulline, symmetric dimethylarginine (SDMA), kynurenine, azelaic acid, and galactaric acid that were positively correlated with albuminuria and negatively with eGFR. Of note, some of the most significantly differently excreted metabolites were not identified. Multiple logistic regression, carried out on identified metabolites, recognized 4 features, namely, aspartic acid, azelaic acid, galactaric acid, and symmetric dimethylarginine (SDMA) as relevant for the model and allowed correct identification of DN patients with about 75% accuracy. Zhang et al. [95] carried out serum metabolomic profiling of 8 DN patients, 33 type 2 diabetes mellitus (T2DM) patients, and 25 healthy volunteers in order to investigate the presence of DN biomarkers. Importantly, they reported significant changes of leucine, dihydrosphingosine and phytoshpingosinewere specifically in the DN cohort, thus suggesting the perturbations of amino acid metabolism and phospholipid metabolism as key events in diabetic disease. Other authors have instead investigated specific subfractions of the metabolome, namely, compounds linked to purine and pyrimidine metabolism, phospholipids, and fatty acids.

Xia et al. [96] standardized an analytical method for analysis and quantification of purine and pyrimidine metabolites in DN patients and matched healthy controls. According to the well-established association of the purine and pyrimidine metabolic pathway with the development of the DN, they could assess that uric acid, xanthine, and adenosine were significantly increased in DN patients (especially in those at stage V according to Mogensen classification) while inosine is reduced probably as a result of the adenosine deaminase inhibition that catalyzes inosine formation from adenosine. Impaired lipid metabolism has been directly associated with T2DM and DN. Several phospholipids (PLs), significantly upregulated or downregulated in disease models, have been already recognized as potential biomarkers of T2DM or DN [138, 139]. Comprehensive and quantitative analysis of plasma PLs, such as phosphatidylethanolamine, phosphatidylglycerol, phosphatidylcholine, phosphatidylinositol, phosphatidylserine, sphingomyelin, and lysophosphatidylcholine, may selectively distinguish T2DM from DN patients [97]. Targeted quantification of the phospholipids revealed proportional decrease of phosphatidylinositol and linear increase of sphingomyelin in DN patients. Although the molecular pathogenetic mechanisms leading to impaired metabolism of phospholipids are not clear, the authors suggest that reduced phosphatidylinositol may reflect increased sorbitol pathway activation in T2DM while increased sphingomyelin may depend on glucocorticoids-mediated sphingolipids metabolism.

Also plasma fatty acids (FAs) may have a direct impact on the occurrence and development of diabetes since their abnormal accumulation in parenchymal cells of multiple tissues, called lipotoxicity, has been suggested as a trigger of T2DM and its chronic complications [140]. Specific metabolomics screening of FAs, namely, lipidomics, may contribute to the understanding of this disease. Han and colleagues reported a standardized method based on Gas Chromatography-Mass Spectrometry (CG-MS) useful for the specific assessment of nonesterified and esterified fatty acids (NEFAs and EFAs, resp.) [98]. Lipidomics screening of 150 patients including diabetics with and without nephropathy showed high discrimination power on different stage of DN. Disease progression was specifically correlated with plasma levels of arachidonic acid that is involved in the anabolism of prostaglandins, thus suggesting a key role of the inflammatory processes in the progression of DN.

## 7. Conclusion

As genetic studies conducted so far are still inconclusive, it is difficult to envisage a common genetic basis for the development of DN. Quite possibly a number of environmental factors contribute significantly toward the evolution of the diabetic patient to this specific complication. However, there is no doubt that, from the earliest stages of the disease, many molecular changes, observed at the transcriptomics, proteomics, and metabolomics level, anticipate the onset of a clinical phenotype and may allow us to reconstruct in detail the pathogenetic basis of kidney damage in T2DM. Although new omics challenges such as the analysis of the protein post-translational modifications and of multiprotein complexes, mimicking what naturally happen in intracellular behavior, will further broaden our understanding of the DN pathogenesis, we are already able to identify the common thread that unites all the disparate molecular changes described in the literature by performing bioinformatic-based analysis of genes, transcripts, proteins, and metabolites described so far. We can envisage that the selection of specific omic biomarkers and clinical phenotypes might lead to a better stratification of patient's specific “type” of renal damage in T2DM and might allow the identification of patients that progress or respond to a specific therapy. To accomplish this task and go forward, however, there is an urgent need to build up disease-specific platforms containing personal, clinical, and omics profiles that will allow the full potential application of systems biology analysis and the development of specific disease phenotype models.

We can expect in the next future the development of new paradigms of renal damage in T2DM that will contribute to defining of the road to the molecular medicine as a global, organized approach applicable to DN as well as to other relevant renal conditions.

## Figures and Tables

**Table 1:** Genetic markers. Collection of significant genetic markers, listed alphabetically.

Nearest gene(s)	Variant	Ethnicity	Diabetes type	Assay type	Potential value of biomarker	References
18q	D18S1364	African American	T2D	Linkage analysis	Diagnostic	[11]
18q22.3-23	D18S43/D18S50	Turkish	T2D	Linkage analysis	Diagnostic	[12]
18q22.3-23	D18S43/D18S50	Pima Indians	T2D	Linkage analysis	Diagnostic	[12]
7p	D7S3051	African American	T2D	Linkage analysis	Diagnostic	[11]
AGTR1	rs12695897	African American	T2D	Candidate based genotyping	Diagnostic	[13]
APOL3	rs16996381	African American	T2D	GWAS	Diagnostic	[14]
AUH	rs773506	African American	T2D	GWAS	Diagnostic	[14]
C12orf66/TMEM5	rs11175885	African American	T2D	GWAS	Diagnostic	[14]
C6orf167	rs3822908	African American	T2D	GWAS	Diagnostic	[14]
C6orf191/ARHGAP18	rs208865	African American	T2D	GWAS	Diagnostic	[14]
CHN2	rs2057737	African American	T2D	Candidate based genotyping	Diagnostic	[13]
CHN2	rs3729621	African American	T2D	Candidate based genotyping	Diagnostic	[13]
CHN2	rs3793313	African American	T2D	Candidate based genotyping	Diagnostic	[13]
CNDP1	D18S880	European	T1D/T2D	Candidate based genotyping	Diagnostic	[15]
CNDP1	D18S880	Multiethnic	T2D	Meta-analysis	Diagnostic	[16]
CNDP1	rs4892249	African American	T2D	Candidate based genotyping	Diagnostic	[13]
CNDP1	rs6566815	African American	T2D	Candidate based genotyping	Diagnostic	[13]
CNDP1	D18S880	Caucasian	T2D	Meta-analysis	Diagnostic	[17]
ELMO1	rs741301	Asian	T2D	Meta-analysis	Diagnostic	[16]
ELMO1	rs11769038	Caucasian	T1D	GWAS	Diagnostic	[18]
ELMO1	rs1882080	Caucasian	T1D	GWAS	Diagnostic	[18]
ELMO1	rs2041801	Caucasian	T1D	GWAS	Diagnostic	[18]
ELMO1	rs7785934	Caucasian	T1D	GWAS	Diagnostic	[18]
ELMO1	Intron 18 + 9170 (A/G)	Japanese	T2D	GWAS	Diagnostic	[19]
FRMD3	rs1535753	African American	T2D	GWAS	Diagnostic	[20]
FRMD3	rs2378658	African American	T2D	GWAS	Diagnostic	[20]
FRMD3	rs942278	African American	T2D	GWAS	Diagnostic	[20]
FRMD3	rs942280	African American	T2D	GWAS	Diagnostic	[20]
FRMD3	rs942283	African American	T2D	GWAS	Diagnostic	[20]
FRMD3	rs1888747	European	T2D	Candidate based genotyping	Diagnostic	[21]
GRIK2	rs7760831	African American	T2D	GWAS	Diagnostic	[14]
GRIP1/CAND1	rs11176482	African American	T2D	GWAS	Diagnostic	[14]
GRIP1/CAND1	rs2904532	African American	T2D	GWAS	Diagnostic	[14]
LIMK2	rs2106294	African American	T2D	GWAS	Diagnostic	[14]
LIMK2	rs4820043	African American	T2D	GWAS	Diagnostic	[14]
MSRB3/HMGA2	rs2358944	African American	T2D	GWAS	Diagnostic	[14]
MYH9	rs735853	African American	T2D	GWAS	Diagnostic	[14]
NAV3	rs12302041	African American	T2D	GWAS	Diagnostic	[14]
ND	rs1978243	African American	T2D	GWAS	Diagnostic	[14]
ND	rs4260465	African American	T2D	GWAS	Diagnostic	[14]
ND	rs7697691	African American	T2D	GWAS	Diagnostic	[14]
OR2L13	rs10888287	African American	T2D	GWAS	Diagnostic	[14]
PVT1	rs2648875	Pima Indians	T2D	GWAS	Diagnostic	[22]
PVT1	rs13447075	European	T1D	Candidate based genotyping	Diagnostic	[23]
RNF185	rs1034589	African American	T2D	GWAS	Diagnostic	[14]
RPS12	rs7769051	African American	T2D	GWAS	Diagnostic	[14]
RPS12	rs9493454	African American	T2D	GWAS	Diagnostic	[14]
SASH1	rs6930576	African American	T2D	GWAS	Diagnostic	[14]
SFI1	rs5749286	African American	T2D	GWAS	Diagnostic	[14]
SLC10A7/LSM6	rs891382	African American	T2D	GWAS	Diagnostic	[14]
TPM1	rs6494387	African American	T2D	GWAS	Diagnostic	[14]
UNC5C	rs11730446	African American	T2D	GWAS	Diagnostic	[14]

**Table 2:** Gene expression markers. Collection of coding RNA transcripts showing deregulation in DN. List is ordered alphabetically. IHC: immunohistochemistry. SAGE: serial analysis of gene expression; NGS: next-generation sequencing.

mRNA transcript	Sample type	Tissue compartment	Expression	Diabetes type	Assay type	Potential value of biomarker	References
ABCA1	Kidney	Whole	Down	T2D	qPCR	Diagnostic/prognostic	[24]
ABCG1	Kidney	Whole	Down	T2D	qPCR	Diagnostic/prognostic	[24]
ACE	Kidney	Whole	Up	T2D	qPCR	Diagnostic	[25]
ACE	Kidney	Glomerular	Up	T2D	qPCR	Diagnostic	[26]
ACE	Urine	Sediment	Up	T2D	qPCR	Diagnostic	[27]
ACE2	Kidney	Glomerular	Down	T2D	qPCR	Diagnostic	[26]
ACTN4	Urine	Sediment	Up	T2D	qPCR	Diagnostic	[28]
ACTN4	Urine	Sediment	Up	T2D	qPCR	Diagnostic/prognostic	[29]
ACTN4	Urine	Sediment	Up	T2D	qPCR	Diagnostic	[27]
ANGPTL2	Kidney	Glomerular	Up	T2D	qPCR/IHC	Diagnostic	[30]
ANKRD56	Kidney	Whole	Down	T2D	qPCR	Diagnostic	[31]
apoE	Kidney	Whole	Down	T2D	qPCR	Diagnostic/prognostic	[24]
AQP1	Kidney	Glomerular	Up	T2D	Array	Diagnostic	[32]
ATG5	Kidney	Whole	Down	T2D	qPCR	Diagnostic	[33]
ATG7	Kidney	Whole	Down	T2D	qPCR	Diagnostic	[34]
ATG8	Kidney	Whole	Down	T2D	qPCR	Diagnostic	[33]
B2M	Kidney	Tubulointerstitial	Up	T1D/T2D	Array/qPCR	Diagnostic/prognostic	[35]
B7-1	Kidney	Whole	Up	T2D	qPCR	Diagnostic	[36]
BECN1	Kidney	Whole	Down	T2D	qPCR	Diagnostic	[33]
BECN1	Kidney	Whole	Down	T2D	qPCR	Diagnostic	[34]
BMP2	Kidney	Glomerular	Down	T2D	Array	Diagnostic	[32]
C3	Kidney	Glomerular	Up	ND	Array	Diagnostic	[37]
CAPN3	Kidney	Glomerular	Up	T2D	Array	Diagnostic	[32]
CCL2	Kidney	Tubulointerstitial	Up	T2D	qPCR/IHC	Diagnostic	[38]
CCL5/RANTES	Kidney	Tubulointerstitial	Up	T1D/T2D	qPCR	Diagnostic/prognostic	[35]
CCR5	Kidney	Tubulointerstitial	Up	T2D	qPCR/IHC	Diagnostic	[38]
CD2AP	Urine	Sediment	Up	T2D	qPCR	Diagnostic/prognostic	
CD36	Kidney	Whole	Up	T2D	qPCR	Diagnostic/prognostic	[24]
CD68	Kidney	Tubulointerstitial	Up	T2D	qPCR/IHC	Diagnostic	[38]
CDH2	Urine	Sediment	Up	T2D	qPCR	Diagnostic/prognostic	[27]
CLIC5	Kidney	Glomerular	Down	ND	Array/IHC	Diagnostic	[37]
COL1A2	Kidney	Tubulointerstitial	Up	T1D/T2D	qPCR	Diagnostic	[39]
COL1A2	Kidney	Glomerular	Up	ND	Array	Diagnostic	[37]
COL1A2	Kidney	Tubular	Up	ND	Array	Diagnostic	[37]
COL3A1	Kidney	Tubular	Up	ND	Array	Diagnostic	[37]
COL4A1	Kidney	Tubulointerstitial	Up	T1D/T2D	qPCR	Diagnostic	[39]
COL4A1	Urine	Sediment	Up	T2D	qPCR	Diagnostic	[27]
COL4A2	Kidney	Tubulointerstitial	Up	T1D/T2D	qPCR	Diagnostic	[39]
COL6A3	Kidney	Glomerular	Up	ND	Array	Diagnostic	[37]
COL8A1	Kidney	Glomerular/tubulointerstitial	Up	T2D	qPCR/IHC	Diagnostic	[40]
COL8A2	Kidney	Glomerular/tubulointerstitial	Up	T2D	qPCR/IHC	Diagnostic	[40]
CTGF	Kidney	Glomerular	Down	T2D	Array/qPCR/IHC	Diagnostic	[41]
CXCL10/IP10	Kidney	Tubulointerstitial	Up	T1D/T2D	Array/qPCR	Diagnostic/prognostic	[35]
CXCL16	Kidney	Whole	Down	T2D	qPCR	Diagnostic/prognostic	[24]
CXCL6	Kidney	Glomerular	Up	ND	Array	Diagnostic	[37]
DKK3	Kidney	Glomerular	Up	T2D	Array	Diagnostic	[42]
EDN1	Kidney	Tubulointerstitial	Up	T1D/T2D	qPCR	Diagnostic/prognostic	[35]
EGF	Kidney	Tubulointerstitial	Down	T1D/T2D	qPCR/IHC	Diagnostic/prognostic	[39]
ENTPD8	Kidney	Whole	Down	T2D	qPCR	Diagnostic	[31]
FAT1	Urine	Sediment	Up	T2D	qPCR	Diagnostic	[27]
FGF-2	Kidney	Tubulointerstitial	Up	T2D	qPCR/IF	Diagnostic	[43]
FN1	Kidney	Tubulointerstitial	Up	T1D/T2D	qPCR	Diagnostic	[39]
FOXO1	Kidney	Whole	Down	T2D	qPCR/IHC	Diagnostic	[33]
FOXO3A	Kidney	Whole	Down	T2D	qPCR	Diagnostic	[33]
FSP1	Kidney	Glomerular	Up	T2D	qPCR/ISH	Diagnostic/prognostic	[44]
GREM1	Kidney	Whole	Up	T2D	qPCR/ISH/IHC	Diagnostic	[45]
GREM1	Kidney	Whole	Up	T2D	qPCR/ISH	Diagnostic/prognostic	[46]
HDAC2	Kidney	Whole	Up	T2D	qPCR/IHC	Diagnostic/prognostic	[34]
HDAC4	Kidney	Whole	Up	T2D	qPCR/IHC	Diagnostic/prognostic	[34]
HDAC5	Kidney	Whole	Up	T2D	qPCR/IHC	Diagnostic/prognostic	[34]
HES1	Kidney	Whole	Up	T2D	qPCR/ISH	Diagnostic	[46]
HLA-A	Kidney	Tubulointerstitial	Up	T1D/T2D	Array/qPCR	Diagnostic/prognostic	[35]
HLA-B	Kidney	Tubulointerstitial	Up	T1D/T2D	Array/qPCR	Diagnostic/prognostic	[35]
HSPA5	Kidney	Tubulointerstitial	Up	T2D	Array/qPCR	Diagnostic	[47]
Hyaluronoglucosidase	Kidney	Glomerular	Up	T2D	Array	Diagnostic	[32]
HYOU1	Kidney	Tubulointerstitial	Up	T2D	Array/qPCR	Diagnostic	[47]
IFNB1	Kidney	Tubulointerstitial	Up	T1D/T2D	qPCR	Diagnostic/prognostic	[35]
IGF-1	Kidney	Glomerular	Down	T2D	Array	Diagnostic	[32]
IGFBP-2	Kidney	Glomerular	Down	T2D	Array	Diagnostic	[32]
IGH	Kidney	Glomerular	Up	ND	Array	Diagnostic	[37]
IGH	Kidney	Tubular	Up	ND	Array	Diagnostic	[37]
IGL	Kidney	Tubular	Up	ND	Array	Diagnostic	[37]
IHG-1	Kidney	Tubulointerstitial	Up	T2D	ISH	Diagnostic	[48]
IL6	Kidney	Tubulointerstitial	Up	T2D	qPCR/IHC	Diagnostic	[38]
IRS2	Kidney	Tubulointerstitial	Up	T2D	ISH	Diagnostic	[49]
JAG1	Kidney	Whole	Up	T2D	qPCR/ISH	Diagnostic	[46]
JAK2	Kidney	Glomerular/tubulointerstitial	Up	T2D	Array/qPCR/IHC	Diagnostic/prognostic	[50]
LC3	Kidney	Whole	Down	T2D	qPCR	Diagnostic	[33]
LC3	Kidney	Whole	Down	T2D	qPCR	Diagnostic	[34]
LDLR	Kidney	Whole	Up	T2D	qPCR	Diagnostic/prognostic	[24]
LEF1	Kidney	Glomerular	Up	T2D	Array	Diagnostic	[42]
LOX1	Kidney	Whole	Up	T2D	qPCR	Diagnostic/prognostic	[24]
MMP14	Kidney	Tubulointerstitial	Up	T1D/T2D	qPCR	Diagnostic	[39]
MMP2	Kidney	Tubulointerstitial	Up	T1D/T2D	qPCR	Diagnostic	[39]
MMP7	Kidney	Tubulointerstitial	Up	ND	Array/qPCR/IHC	Diagnostic	[51]
MMP7	Kidney	Tubulointerstitial	Up	T1D/T2D	qPCR	Diagnostic	[39]
MRP8	Kidney	Glomerular	Up	T2D	qPCR/IHC	Diagnostic/prognostic	[52]
NOTCH3	Urine	Sediment	Up	T2D	qPCR	Diagnostic	[27]
NPHS1	Kidney	Glomerular	Down	T2D	Array	Diagnostic	[41]
NPHS1	Kidney	Glomerular	Down	T2D	Array/IHC	Diagnostic	[32]
NPHS1	Urine	Sediment	Up	T2D	qPCR	Diagnostic	[53]
NPHS1	Kidney	Whole	Down	T2D	ISH	Diagnostic/prognostic	[54]
NPHS1	Urine	Sediment	Up	T2D	qPCR	Diagnostic/prognostic	[28]
NPHS1	Kidney	Glomerular	Down	ND	Array	Diagnostic	[37]
NPHS2	Kidney	Glomerular	Down	T2D	Array	Diagnostic	[41]
NPHS2	Urine	Sediment	Up	T2D	qPCR	Diagnostic	[53]
NPHS2	Urine	Sediment	Up	T2D	qPCR	Diagnostic/prognostic	[28]
NPHS2	Kidney	Glomerular	Down	ND	Array/IHC	Diagnostic/prognostic	[37]
NPHS2	Urine	Sediment	Up	T2D	qPCR	Diagnostic	[29]
NRP1	Kidney	Glomerular	Down	T2D	qPCR	Diagnostic	[55]
NRP2	Kidney	Glomerular	Down	T2D	qPCR	Diagnostic	[55]
OPG	Kidney	Tubulointerstitial	Up	T2D	Array/qPCR	Diagnostic/prognostic	[56]
PDGF-A	Kidney	Whole	Up	T2D	qPCR/IHC	Diagnostic	[57]
PDGF-B	Kidney	Whole	Up	T2D	qPCR/IHC	Diagnostic	[57]
PECAM-1	Kidney	Glomerular	Up	T2D	Array	Diagnostic	[32]
PKC*α*	Kidney	Glomerular	Up	T2D	qPCR/IHC	Diagnostic	[58]
PLA2R1	Kidney	Glomerular	Down	ND	Array	Diagnostic	[37]
PLCE1	Kidney	Glomerular	Down	ND	Array	Diagnostic	[37]
PODXL	Kidney	Glomerular	Down	ND	Array	Diagnostic	[37]
PODXL	Urine	Sediment	Up	T2D	qPCR	Diagnostic/prognostic	[29]
PRKCB	Kidney	Whole	Up	T2D	qPCR	Diagnostic	[59]
PTGDS	Kidney	Glomerular	Down	ND	Array	Diagnostic	[37]
ROBO2	Kidney	Glomerular	Down	T2D	qPCR	Diagnostic	[60]
SMPDL3b	Kidney	Glomerular	Up	T2D	Array	Diagnostic	[61]
STAT1	Kidney	Whole	Up	T2D	qPCR/IHC	Diagnostic	[33]
SYNPO	Urine	Sediment	Up	T2D	qPCR	Diagnostic	[28]
SYNPO	Kidney	Glomerular	Down	ND	Array	Diagnostic	[37]
SYNPO	Urine	Sediment	Up	T2D	qPCR	Diagnostic/prognostic	[29]
SYNPO	Urine	Sediment	Up	T2D	qPCR	Diagnostic	[27]
TIMP1	Kidney	Tubulointerstitial	Up	T1D/T2D	qPCR	Diagnostic	[39]
TIMP1	Urine	Sediment	Down	T2D	qPCR	Diagnostic	[27]
TIMP3	Kidney	Whole	Down	T2D	qPCR/IHC	Diagnostic	[33]
TIMP3	Kidney	Tubulointerstitial	Up	T1D/T2D	qPCR	Diagnostic	[39]
TIPE2	Kidney	Whole	Up	T2D	qPCR/WB	Diagnostic	[62]
TLR4	Kidney	Glomerular/tubulointerstitial	Up	T2D	qPCR/IHC	Diagnostic/prognostic	[38]
TNFAIP8	Kidney	Whole	Up	T2D	qPCR/WB	Diagnostic	[62]
TRAIL	Kidney	Tubulointerstitial	Up	T2D	Array/IHC/qPCR	Diagnostic/prognostic	[56]
TWIST1	Urine	Sediment	Up	T2D	qPCR	Diagnostic	[27]
UII	Kidney	Whole	Up	T2D	qPCR/IHC	Diagnostic	[63]
UT	Kidney	Whole	Up	T2D	qPCR	Diagnostic	[63]
VCAM1	Kidney	Tubulointerstitial	Up	T1D/T2D	Array/qPCR	Diagnostic/prognostic	[35]
VEGF	Kidney	Glomerular	Down	T2D	Array/qPCR/IHC	Diagnostic	[41]
VEGF	Kidney	Glomerular	Down	T2D	Array/IHC	Diagnostic/prognostic	[32]
VEGF	Kidney	Glomerular	Down	T2D	qPCR	Diagnostic/prognostic	[64]
VEGF	Kidney	Whole	Up	T2D	ISH/IHC	Diagnostic	[65]
VEGF	Kidney	Tubulointerstitial	Down	T1D/T2D	qPCR/IHC	Diagnostic/prognostic	[39]
WNT1	Kidney	Glomerular	Up	T2D	Array	Diagnostic	[42]
WNT16	Kidney	Glomerular	Up	T2D	Array	Diagnostic	[42]
WNT2B	Kidney	Glomerular	Up	T2D	Array	Diagnostic	[42]
WNT4	Kidney	Glomerular	Up	T2D	Array	Diagnostic	[42]
WNT6	Kidney	Glomerular	Up	T2D	Array	Diagnostic	[42]
WT1	Kidney	Glomerular	Down	T2D	Array/IHC	Diagnostic	[41]
WT1	Urine	Sediment	Up	T2D	qPCR	Diagnostic	[28]
WT1	Kidney	Glomerular	Down	ND	Array	Diagnostic/prognostic	[37]
XBP1	Kidney	Tubulointerstitial	Up	T2D	Array/qPCR	Diagnostic	[47]

**Table 3:** Noncoding RNA markers. Collection of noncoding RNA transcripts deregulated in DN samples. List is ordered alphabetically.

miRNA transcript	Sample type	Expression	Assay type	Diabetes type	Potential value of biomarker	References
hsa-miR-1205	Mesangial cells	Up	qPCR	—	Descriptive	[66]
hsa-miR-129	Human mesangial cells	Up	Array/qPCR	—	—	[67]
hsa-miR-130a	Urinary exosomes	Up	qPCR	T1D	Diagnostic	[68]
hsa-miR-145	Urinary exosomes	Up	qPCR	T1D	Diagnostic	[68]
hsa-miR-146a	Kidney	Up	Array/qPCR	T2D	Diagnostic	[69]
hsa-miR-15	Urinary sediment	Down	qPCR	—	Diagnostic/prognostic	[70]
hsa-miR-155	Urinary exosomes	Down	qPCR	T1D	Diagnostic/prognostic	[68]
hsa-miR-155	Kidney	Up	Array/qPCR	T2D	Prognostic	[69]
hsa-miR-188-3p	Urine	Down	qPCR	T1D	Prognostic	[71]
hsa-miR-1913	Urine	Up	qPCR	T1D	Prognostic	[71]
hsa-miR-192	Kidney	Down	qPCR/ISH	—	Diagnostic/prognostic	[72]
hsa-miR-192	Human mesangial cells	Up	Array	—	—	[67]
hsa-miR-192	Urinary sediment	Down	qPCR	—	Diagnostic/prognostic	[73]
hsa-miR-21	Kidney	Up	qPCR	T2D	Diagnostic	[74]
hsa-miR-214-3p	Urine	Up	qPCR	T1D	Prognostic	[71]
hsa-miR-221-3p	Urine	Down	qPCR	T1D	Prognostic	[71]
hsa-miR-29a	Urine	Up	qPCR	T2D	Prognostic	[75]
hsa-miR-323b-5p	Urine	Down	qPCR	T1D	Prognostic	[71]
hsa-miR-337	Human mesangial cells	Up	Array/qPCR	—	—	[67]
hsa-miR-373-5p	Urine	Up	qPCR	T1D	Prognostic	[71]
hsa-miR-377	Human mesangial cells	Up	Array/qPCR	—	—	[67]
hsa-miR-424	Urinary exosomes	Down	qPCR	T1D	Prognostic	[68]
hsa-miR-429	Urine	Up	qPCR	T1D	Prognostic	[71]
hsa-miR-524-5p	Urine	Down	qPCR	T1D	Prognostic	[71]
hsa-miR-638	Urine	Up	qPCR	T1D	Prognostic	[71]
hsa-miR-765	Urine	Up	qPCR	T1D	Prognostic	[71]
hsa-miR-92b-5p	Urine	Up	qPCR	T1D	Prognostic	[71]
let-7a	Whole blood	Down	Array/qPCR	T2D	Diagnostic	[76]
PVT1 (lncRNA)	Mesangial cells	Up	qPCR	—	Descriptive	[66]

**Table 4:** Epigenetic markers. List of epigenetic marks identified in DN. List is ordered alphabetically.

Locus	Sample type	Type of modification	Diabetes type	Potential value of biomarker	References
SHC1	PBMC	Reduced promoter methylation	—	Diagnostic	[77]
UNC13B	Whole blood	Increased DNA methylation	T1D	Diagnostic/prognostic	[78]

**Table 5:** Proteomic biomarkers. List of significant protein biomarkers ordered as they are cited in the text. LCM: Laser Capture Microdissection; LC/MS/MS: liquid chromatography coupled to tandem mass spectrometry; IF: immunofluorescence; IHC: immunohistochemistry; SELDI-TOF/MS: Surface Enhanced Laser Desorption Ionization Mass Spectrometry; CE-MS: capillary electrophoresis; 2DE: two-dimensional electrophoresis.

Protein	Code	Sample	Expression	Assay type	Diabetes type	Potential value of biomarker	References
Integrin, alpha 1	ITGA1	FFPE Kidney	Up	LCM + LC/MS/MS; IHC	T2D	Diagnostic	[79]
Laminin, beta 2	LAMB2, LAMS	FFPE Kidney	Up	LCM + LC/MS/MS; IHC	T2D	Diagnostic	[79]
Nephronectin	NPNT, EGFL6L, POEM, and UNQ295/PRO334	FFPE Kidney	Up	LCM + LC/MS/MS; IHC	T2D	Diagnostic	[79]
Actinin, alpha 4	ACTN4	FFPE Kidney	Up	LCM + LC/MS/MS; IHC	T2D	Diagnostic	[79]
C3	C3, CPAMD1	FFPE Kidney	Up	LCM + LC/MS/MS; IF	T2D	Diagnostic	[80]
C5b-9	C5, CPAMD4	FFPE Kidney	Up	LCM + LC/MS/MS; IF	T2D	Diagnostic	[80]
Fibrinogen *α*-chain	FGA	FFPE Kidney	Up	LCM + LC/MS/MS; IF	T2D	Diagnostic	[80]
Synaptopodin	SYNPO, KIAA1029	FFPE Kidney	Up	LCM + LC/MS/MS; IF	T2D	Diagnostic	[80]
Collagen *α*-1 (I) chain	CO1A1_HUMAN	Urine	Down	CE-MS	T2D	Prognostic	[81]
Collagen *α*-1 (III) chain	CO3A1_HUMAN	Urine	Down	CE-MS	T2D	Prognostic	[81]
Collagen *α*-2 (I) chain	CO1A2_HUMAN	Urine	Down	CE-MS	T2D	Prognostic	[81]
Neurosecretory protein VGF	VGF_HUMAN	Urine	Down	CE-MS	T2D	Prognostic	[81]
Osteopontin	OSTP_HUMAN	Urine	Down	CE-MS	T2D	Prognostic	[81]
Polymeric immunoglobulin receptor	PIGR_HUMAN	Urine	Down	CE-MS	T2D	Prognostic	[81]
Serum albumin	ALBU_HUMAN	Urine	Up	CE-MS	T2D	Prognostic	[81]
Sodium/potassium-transporting ATPase *γ* chain	ATNG_HUMAN	Urine	Down	CE-MS	T2D	Prognostic	[81]
Pro-SAAS	PCSK1_HUMAN	Urine	Up	CE-MS	T2D	Prognostic	[81]
*α*-2-HS-glycoprotein	FETUA_HUMAN	Urine	Up	CE-MS	T2D	Prognostic	[81]
a-1 acid glycoprotein	AGP	Urine	Up	SDS-PAGE + LC/MS/MS + ELISA	T1D	Prognostic	[82]
Clusterin	CLU, APOJ, CLI, KUB1, and AAG4	Urine	Comparable	SDS-PAGE + LC/MS/MS + ELISA	T1D	Prognostic	[82]
Progranulin	GRN	Urine	Up	SDS-PAGE + LC/MS/MS + ELISA	T1D	Prognostic	[82]
Tamms-Horsfall glycoprotein	THP	Urine	Up	SDS-PAGE + LC/MS/MS + ELISA	T1D	Prognostic	[82]
Alpha-1-acid glycoprotein 1	ORM1, AGP1	Urine	Up	iTRAQ labelling + LC-MS/MS; WB	T2D	Diagnostic	[83]
Alpha-1-antitrypsin	SERPINA1, AAT, PI, PRO0684, and PRO2209	Urine	Up	iTRAQ labelling + LC-MS/MS; WB	T2D	Diagnostic	[83]
Prostate stem cell antigen	PSCA, UNQ206/PRO232	Urine	Up	iTRAQ labelling + LC-MS/MS; WB	T2D	Diagnostic	[83]
Ubiquitin ribosomal fusion protein (UbA52)	UBA52, UBCEP2	Urine	Up	SELDI-TOF/MS, WB	T2D	Diagnostic	[84]
*β*2-microglobulin	B2M, CDABP0092, and HDCMA22P	Urine	Up	SELDI-TOF/MS, WB, ELISA	T2D	Diagnostic	[84, 85]
Free ubiquitin	UBB, UBC, UBA52, and RPS27A	Urine	Up	SELDI-TOF/MS, WB, ELISA	T2D	Diagnostic	[85]
Histone-lysine N-methyltransferase 2C	KMT2C, HALR, KIAA1506, and MLL3	Urine exosomes	Up	2DE + LC/MS/MS	T2D	Diagnostic	[86]
Voltage-dependent anion-selective channel protein 1	VDAC1, VDAC	Urine exosomes	Down	2DE + LC/MS/MS	T2D	Diagnostic	[86]
Alpha-1-microglobulin/ bikunin precursor	AMBP, HCP, and ITIL	Urine exosomes	Up	2DE + LC/MS/MS	T2D	Diagnostic	[86]
Vasorin (glycated)	VASN, SLITL2, UNQ314/PRO357/ PRO1282	Plasma	Up	LC/MS/MS; WB	T2D	Diagnostic	[87]
Retinol binding protein-4 (glycated)	RBP4, and PRO2222	Plasma	Up	LC/MS/MS; WB	T2D	Diagnostic	[87]
Lumican (glycated)	LUM, LDC, SLRR2D	Plasma	Up	LC/MS/MS; WB	T2D	Diagnostic	[87]
Vasorin (glycated)	VASN, SLITL2, and UNQ314/PRO357/ PRO1282	Plasma	Up	LC/MS/MS; WB	T2D	Diagnostic	[87]
Hemopexin precursor (glycated)	MMP15	Plasma	Up	2DE; ESI-Q-TOF/MS/MS	T2D	Diagnostic	[88]
Alpha-1-antitrypsin (glycated)	SERPINA1, AAT, PI, PRO0684, and PRO2209	Plasma	Up	2DE; ESI-Q-TOF/MS/MS	T2D	Diagnostic	[88]
Haptoglobin-related protein (glycated)	HPR	Plasma	Up	2DE; ESI-Q-TOF/MS/MS	T2D	Diagnostic	[88]
Serine proteinase inhibitor (glycated)	SERPINA5, PCI, PLANH3, and PROCI	Plasma	Up	2DE; ESI-Q-TOF/MS/MS	T2D	Diagnostic	[88]
Complement factor C4B3 (glycated)	C4B, CO4, CPAMD3, and C4B_2	Plasma	Up	2DE; ESI-Q-TOF/MS/MS	T2D	Diagnostic	[88]
Prekallikrein (glycated)	KLKB1, KLK3	Plasma	Up	2DE; ESI-Q-TOF/MS/MS	T2D	Diagnostic	[88]
Apolipoprotein (ApoE)	APOE	Plasma	Up	2DE; MALDI-TOF/MS/MS	T2D	Diagnostic	[89]
Glutathione peroxidase (eGPx)	GPX2	Plasma	Up	2DE; MALDI-TOF/MS/MS	T2D	Diagnostic	[89]
Vitamin D-binding protein (DBP)	GC	Plasma	Up	2DE; MALDI-TOF/MS/MS	T2D	Diagnostic	[89]

**Table 6:** Metabolomics biomarkers. List of significant metabolites ordered as they are cited in the text. LC/MS/MS: liquid chromatography coupled to tandem mass spectrometry; GC-MS: gas chromatography/mass spectrometry; NMR: Nuclear Magnetic Resonance; CE-MS: capillary electrophoresis; HPLC–UV/MS/MS: high pressure liquid chromatography coupled to UV and mass spectrometry; UPLC–oa-TOF-MS: ultra performance liquid chromatography coupled to time of flight mass spectrometry.

Metabolite	Code	Sample	Expression	Assay type	Diabetes type	Potential value of biomarker	References
2-Methyl acetoacetate	HMDB03771	Urine	Down	GC-MS	T2D	Diagnostic	[90]
3-Methyl adipic acid	HMDB00555	Urine	Down	GC-MS	T2D	Diagnostic	[90]
3-Methyl crotonyl glycine	HMDB00459	Urine	Down	GC-MS	T2D	Diagnostic	[90]
2-Ethyl 3-OH propionate	HMDB00396	Urine	Down	GC-MS	T2D	Diagnostic	[90]
3-Hydroxyisobutyrate	HMDB00435	Urine	Down	GC-MS	T2D	Diagnostic	[90]
3-Hydroxyisovalerate	HMDB00754	Urine	Down	GC-MS	T2D	Diagnostic	[90]
3-Hydroxypropionate	HMDB00700	Urine	Down	GC-MS	T2D	Diagnostic	[90]
Aconitic acid	HMDB00958	Urine	Down	GC-MS	T2D	Diagnostic	[90]
Citric acid	HMDB00094	Urine	Down	GC-MS	T2D	Diagnostic	[90]
Glycolic acid	HMDB00115	Urine	Down	GC-MS	T2D	Diagnostic	[90]
Homovanillic acid	HMDB00118	Urine	Down	GC-MS	T2D	Diagnostic	[90]
Tiglylglycine	HMDB00959	Urine	Down	GC-MS	T2D	Diagnostic	[90]
Uracil	HMDB00300	Urine	Down	GC-MS	T2D	Diagnostic	[90]
Butenoylcarnitine	HMDB13126	Plasma	Up	GC-MS	T2D	Prognostic	[91]
Glutamine	HMDB00641	Urine	Down	GC-MS	T2D	Prognostic	[91]
Hexose	HMDB33704	Urine	Down	GC-MS	T2D	Prognostic	[91]
Histidine	HMDB00177	Plasma	Down	GC-MS	T2D	Prognostic	[91]
Tyrosine	HMDB00158	Urine	Down	GC-MS	T2D	Prognostic	[91]
Hippuric acid	HMDB00714	Urine	Down	LC-MS	T1D	Prognostic	[92]
S-(3-Oxododecanoyl) cysteamine	HMDB59773	Urine	Up	LC-MS	T1D	Prognostic	[92]
Substituted carnitine	HMDB00062	Urine	Up	LC-MS	T1D	Prognostic	[92]
3-OH-isovalerate	HMDB00754	Serum	Down	NMR	T2D	Diagnostic	[93]
4-Aminobutyrate + (CH-CH2-CH2-)	HMDB00112	Serum	Up	NMR	T2D	Diagnostic	[93]
Alanine	HMDB00161	Serum	Down	NMR	T2D	Diagnostic	[93]
Cholesterol	HMDB00067	Serum	Down	NMR	T2D	Diagnostic	[93]
Choline	HMDB00097	Serum	Down	NMR	T2D	Diagnostic	[93]
Creatine	HMDB00064	Serum	Down	NMR	T2D	Diagnostic	[93]
Creatine-P	HMDB01511	Serum	Down	NMR	T2D	Diagnostic	[93]
Creatinine	HMDB00562	Serum	Up	NMR	T2D	Diagnostic	[93]
Dimethylamine	HMDB00087	Serum	Down	NMR	T2D	Diagnostic	[93]
Glucose	HMDB00122	Serum	Up	NMR	T2D	Diagnostic	[93]
Glutamine	HMDB00641	Serum	Down	NMR	T2D	Diagnostic	[93]
Isoleucine	HMDB00172	Serum	Down	NMR	T2D	Diagnostic	[93]
Isoleucine	HMDB00172	Serum	Down	NMR	T2D	Diagnostic	[93]
Lactate	HMDB00190	Serum	Up	NMR	T2D	Diagnostic	[93]
Leucine	HMDB00687	Serum	Up	NMR	T2D	Diagnostic	[93]
Leucine + isoleucine	HMDB28932	Serum	Down	NMR	T2D	Diagnostic	[93]
Lipid (-CH3)	—	Serum	Up	NMR	T2D	Diagnostic	[93]
Lipids (beta-CH2)	—	Serum	Up	NMR	T2D	Diagnostic	[93]
Lipids (CH2-)n	—	Serum	Up	NMR	T2D	Diagnostic	[93]
N-Acetylglutamine	HMDB06029	Serum	Down	NMR	T2D	Diagnostic	[93]
O-Phosphocholine	HMDB01565	Serum	Down	NMR	T2D	Diagnostic	[93]
Proline	HMDB00162	Serum	Down	NMR	T2D	Diagnostic	[93]
Pyruvate	HMDB00243	Serum	Down	NMR	T2D	Diagnostic	[93]
Trimethylamine	HMDB00906	Serum	Down	NMR	T2D	Diagnostic	[93]
Valine	HMDB00883	Serum	Down	NMR	T2D	Diagnostic	[93]
Valine + isoleucine	HMDB29130	Serum	Down	NMR	T2D	Diagnostic	[93]
Aspartic acid	HMDB00191	Serum	Up	CE-MS	T2D	Diagnostic	[94]
Azelaic acid	HMDB00784	Serum	Down	CE-MS	T2D	Diagnostic	[94]
Galactaric acid	HMDB00639	Serum	Down	CE-MS	T2D	Diagnostic	[94]
Symmetric dimethylarginine (SDMA)	HMDB03334	Serum	Up	CE-MS	T2D	Diagnostic	[94]
Dihydrosphingosine	HMDB00269	Serum	Down	UPLC–oa-TOF-MS	T2D	Diagnostic	[95]
Leucine	HMDB00687	Serum	Down	UPLC–oa-TOF-MS	T2D	Diagnostic	[95]
Phytosphingosine	HMDB04610	Serum	Down	UPLC–oa-TOF-MS	T2D	Diagnostic	[95]
Adenosine	HMDB00050	Plasma	Up	HPLC–UV/MS/MS	T2D	Diagnostic	[96]
Creatinine	HMDB00562	Plasma	Up	HPLC–UV/MS/MS	T2D	Diagnostic	[96]
Inosine	HMDB00195	Plasma	Down	HPLC–UV/MS/MS	T2D	Diagnostic	[96]
Uric acid	HMDB00289	Plasma	Up	HPLC–UV/MS/MS	T2D	Diagnostic	[96]
Xanthine	HMDB00292	Plasma	Up	HPLC–UV/MS/MS	T2D	Diagnostic	[96]
Phosphatidylinositol	HMDB06953	Plasma	Down	LC/MS/MS	T2D	Diagnostic	[97]
Sphingomyelin	HMDB12089	Plasma	Up	LC/MS/MS	T2D	Diagnostic	[97]
Arachidonic acid	HMDB01043	Plasma	Up	CG-MS	T2D	Prognostic	[98]
